# Applying ZCT to Two-Phase Boost Converter with IGBT Switches Used

**DOI:** 10.3390/mi13122055

**Published:** 2022-11-24

**Authors:** Kuo-Ing Hwu, Pei-Ching Tseng

**Affiliations:** Department of Electrical Engineering, National Taipei University of Technology, 1, Sec. 3, Zhongxiao E. Rd., Taipei 10608, Taiwan

**Keywords:** current balance, interleaved, IGBT, two-phase boost converter

## Abstract

A zero-current-transition (ZCT) strategy is proposed herein. This strategy is applied to a two-phase boost converter with isolated gate bipolar transistors (IGBTs) used as main switches. However, IGBTs have a current tail during the switch-off interval. Consequently, the proposed constant-frequency ZCT strategy along with common-ground auxiliary switches is employed to decrease the switching loss generated by the current tail. Furthermore, the light-load efficiency can be upgraded by regulating the switch-off instants and switch-on times of the two auxiliary switches. Moreover, two phases are interleaved with one phase having a phase difference of 180° from the other phase, and controlled by a current-sharing controller so that the input current can be distributed between the two phases as evenly as possible. Moreover, only one current sensing circuit is required to obtain information on currents in the two main switches. Above all, the number of phases can be extended with easy control of the ZCT and current balance.

## 1. Introduction

In recent years, due to the impact of the energy crisis, power supply products must have several features, such as power saving, stability and small size, etc. To meet these features, switching power supply will become the trend in power supply development in the future. However, due to the parasitic inductance and parasitic capacitance of the power switch elements in the traditional switching power supply, the power switch will cause switching loss under operation [1]. In addition, if the insulated gate bipolar transistor (IGBT) is used as a power switch, the current tail during the turn-off period will increase the switching loss.

Based on the above, the literature [2,3,4] uses the resonant zero-current switching (ZCS) technique to reduce the switching loss due to hard switching, but the corresponding power switch must withstand extremely high resonant currents, thereby increasing the conduction loss. In addition, since the resonant inductor and the resonant capacitor have a fixed execution time, the ZCS technique requires the use of variable frequency control, so the filter is not easy to design. In the literature [5,6,7,8,9], ZCS techniques with pulse width modulation (PWM) are used. Although this PWM ZCS technique has improved the problem of increased conduction loss and difficulty in filter design, the power switch is still subjected to extremely high resonant inductor currents. In addition, there are many additional components used in [9].

To conquer the shortcomings of the PWM ZCS technique, the zero-current transition (ZCT) [10,11,12] can be realized by fixed frequency control and does not need to increase the current stress of the power switch, thereby reducing the conduction loss and making the filter design easy. In [12], there is a floating auxiliary switch used.

In the following, more papers with a ZCT turn-off are described. The literature [13] starts with the introduction of the conventional ZCT converter. The advantage of this circuit is that it can realize the ZCT turn-off of the main switch without increasing the voltage stress of the main switch, but the disadvantage is that the main switch is hard-switched during the turn-on period, increasing the current stress of the main switch, while the auxiliary switch is also hard-switched during the turn-on and -off period. In [14], a novel improvement is provided by putting a ZCT auxiliary circuit on the secondary side of the circuit, so that the main switch can achieve a ZCT turn-off, although the main switch has no additional current stress during the turn-on period, but on the contrary, the main switch has additional current stress during the turn-off period. The main switch in [15] has no additional current stress during the turn-on period, but the drawback is that the auxiliary switch is floating, which increases the design difficulty of the drive circuit to some extent. In [16], an upgraded method is used to direct the inductive energy that causes the auxiliary switch to have additional voltage stress to the output side through the transformer, and both the main switch and auxiliary switch can achieve a ZCT turn-off, which makes the efficiency improve effectively, but the demerit is that the circuit is complex and has too many switching elements, which increases the difficulty of circuit analysis. In [17], although the circuit is simple and there is no additional current stress on the main switch during the turn-on period, the disadvantage is that the voltage stress on the main switch and auxiliary switch is too large, which requires the use of high voltage withstanding IGBTs and hence increases the cost.

On the other hand, many power supply products nowadays take multiphase interleaved control [18,19,20] to increase the output power. However, the line impedance of each phase is not equal. Consequently, it is necessary to add current sharing control [21,22,23] to distribute the load current evenly in each phase. Furthermore, since the current sharing control should sample the current of each phase, a traditional multi-phase interleaved power supply should have a current sampling circuit for each phase to feedback the current signal of each phase so that the load current can be equally distributed in each phase.

In this paper, we propose a two-phase interleaved boost converter with main IGBT switches having the ZCT turn-on by using the proposed auxiliary circuits, along with the proposed current sensing technique. The basic operating principle and mathematical deduction for the proposed topology are described in detail, then its feasibility is evaluated by the IsSpice software, and finally, a physical prototype, with the FPGA chip utilized as a system control kernel, is provided to demonstrate its effectiveness.

## 2. Proposed Converter

**Figure 1:**
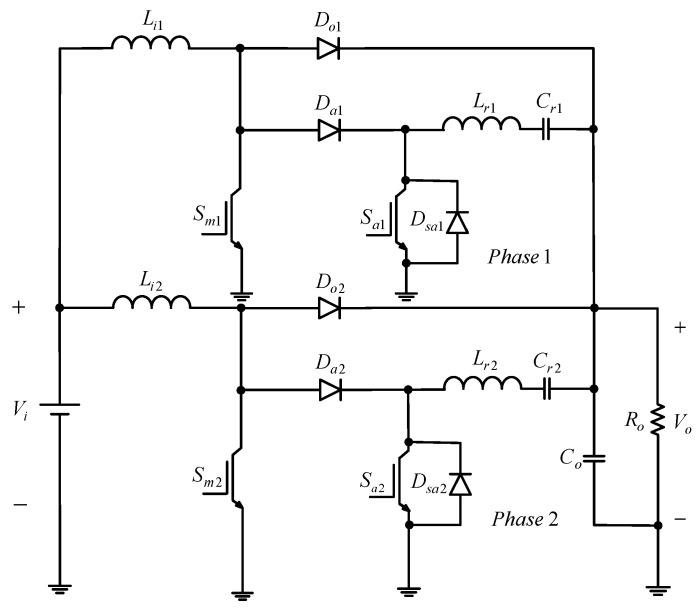
Proposed converter.

Figure 1 shows the proposed two-phase interleaved converter with a new zero current transition. Since this paper adopts the Interleaved PWM technique, the respective duty cycle of one phase differs by 180° from that of the other and does not overlap with each other. Accordingly, the operating principle of the single-phase circuit will be analyzed.

For analysis convenience of analyzing the proposed converter in each operating state, the following assumptions are made first: (i) The active power switch and the passive power switch are ideal; (ii) the input current is constant and the input inductance is much larger than the resonance inductance, which can be considered as a constant current source; (iii) the output voltage is fixed and the output capacitance is much larger than the resonant capacitance, so it can be regarded as a constant voltage source and (iv) Both the capacitance and inductance have no parasitic resistance.

**Figure 2:**
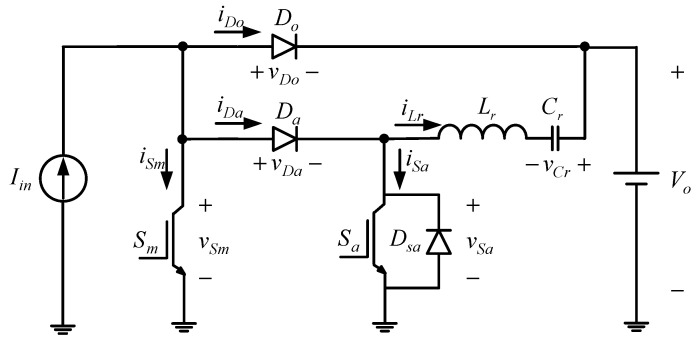
Single-phase equivalent circuit.

From the above assumptions, the single-phase equivalent circuit will be shown in Figure 2.

Prior to the circuit analysis shown in Figure 2, the relevant component symbols are defined as follows: (i) *V_o_* is the output DC voltage, *v_gm_* is the gate signal of the main switch *S_m_*, *v_Sm_*, is the voltage on the main switch, *v_ga_* is the gate signal of the common-grounded auxiliary switch *S_a_*, *v_Sa_* is the voltage on the auxiliary switch, *v_Da_* is the voltage on the auxiliary diode *D_a_*, *v_Do_* is the voltage on the output diode *D_o_*, and *v_Cr_* is the voltage on the resonant capacitor *C_r_* and (ii) *I_in_* is the DC current flowing through the input inductor, *i_Sm_* is the current flowing through the main switch *S_m_*, *i_Sa_* is the current flowing through the auxiliary switch *S_a_*, *i_Da_* is the current flowing through the auxiliary diode *D_a_*, *i_Do_* is the current flowing through the output diode *D_o_*, and *i_Lr_* is the current flowing through the resonant inductor *L_r_*. The internal diode *D_sa_* is connected in parallel with *S_a_*.

**Figure 3:**
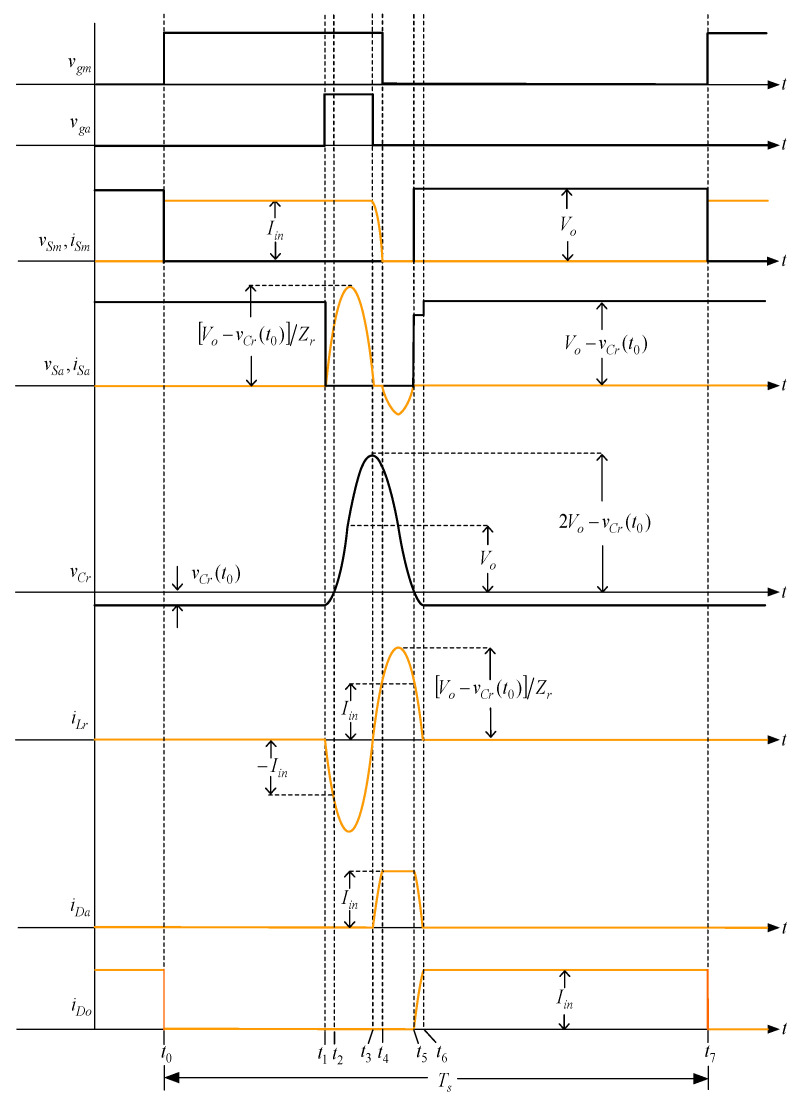
Main operating waveforms.

According to the on/off situation of the switching elements and the state of the energy storage elements, the main operating waveforms of the converter, shown in Figure 3, can be divided into seven over one switching cycle.

**Figure 4:**
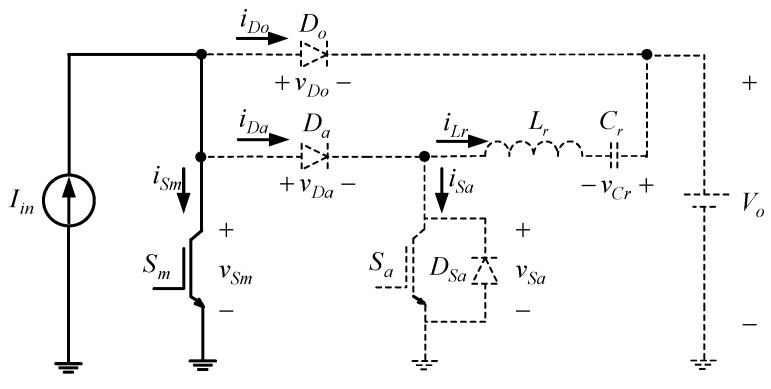
Current flow of state 1.

State 1 [$t_{0}\leq t\leq t_{1}$]: As shown in Figure 3 and Figure 4, when the time reaches *t*_0_, the main switch *S_m_* is on and the rest of the switch elements are in the off state. At the same time, the input current *I_in_* flows through *S_m_*.

According to Figure 3, the following equations can be obtained as

(1) $$\left\{ \begin{array}{l} i_{Lr}(t)=0\\ v_{Cr}(t)=v_{Cr}(t_{0}) \end{array}\right.$$

The time experienced in this state, called Δ*t*_1_, is

(2) $$\Delta t_{1}=t_{1}-t_{0}=DT_{s}-\Delta t_{2}-\Delta t_{3}-\Delta t_{4}$$

where Δ*t*_2_ is the time experienced in state 2, Δ*t*_3_ is the time experienced in state 3, Δ*t*_4_ is the time experienced in state 4, and *D* is the duty cycle.

**Figure 5:**
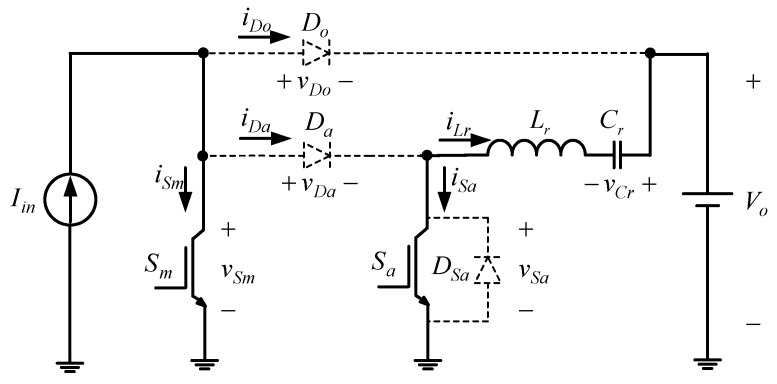
Current flow of state 2.

State 2 [$t_{1}\leq t\leq t_{2}$]: As shown in Figure 3 and Figure 5, when the time reaches *t*_1_, the auxiliary switch *S_a_* is turned on, and the output voltage *V_o_*, resonant inductor *L_r_*, and resonant capacitor *C_r_* form a resonant circuit. At the same time, the resonant capacitor voltage *v_Cr_* rises gradually from *v_Cr_*(*t*_0_), and the resonant inductor current *i_Lr_* starts to fall from zero. Once the voltage *v_Cr_* rises to zero, the operating state proceeds to state 3.

The initial conditions of this state are

(3) $$\left\{ \begin{array}{l} i_{Lr}(t_{1})=0\\ v_{Cr}(t_{1})=v_{Cr}(t_{0}) \end{array}\right.$$

According to Figure 3, the following equations can be obtained as

(4) $$\left\{ \begin{array}{l} i_{Lr}(t)=\frac{v_{Cr}(t_{0})-V_{o}}{Z_{r}}\sin\omega_{r}(t-t_{1})\\ v_{Cr}(t)=V_{o}+\left[v_{Cr}(t_{0})-V_{o}\right]\cos\omega_{r}(t-t_{1}) \end{array}\right.$$

The time experienced in this state, called Δ*t*_2_, is

(5) $$\Delta t_{2}=t_{2}-t_{1}=\frac{1}{\omega_{r}}\sin^{-1}\left(\frac{-Z_{r}I_{in}}{v_{Cr}(t_{0})-V_{o}}\right)$$

State 3 [$t_{2}\leq t\leq t_{3}$]: As shown in Figure 3 and Figure 5, when the time reaches *t*_2_, the resonant inductor *L_r_* and resonant capacitor *C_r_* resonate continuously, and the resonant capacitor voltage *v_Cr_* rises from zero, whereas the resonant inductor current *i_Lr_* decreases continuously. When the resonant capacitor voltage *v_Cr_* is equal to the output voltage *V_o_*, the resonant inductor current *i_Lr_* climbs upward from the reverse peak current. Once the resonant inductor current *i_Lr_* climbs to zero, the operating state enters state 4.

The initial conditions of this state are

(6) $$\left\{ \begin{array}{l} i_{Lr}(t_{2})=-I_{in}\\ v_{Cr}(t_{2})=0 \end{array}\right.$$

According to Figure 3, the following equations can be obtained to be

(7) $$\left\{ \begin{array}{l} i_{Lr}(t)=-I_{in}\cos\omega_{r}(t-t_{2})-\frac{V_{o}}{Z_{r}}\sin\omega_{r}(t-t_{2})\\ v_{Cr}(t)=V_{o}-V_{o}\cos\omega_{r}(t-t_{2})+Z_{r}I_{in}\sin\omega_{r}(t-t_{2}) \end{array}\right.$$

The time experienced in this state, called Δ*t*_3_, is

(8) $$\Delta t_{3}=t_{3}-t_{2}=\frac{1}{\omega_{r}}\cos^{-1}\left(\frac{v_{Cr}(t_{0})V_{o}-V_{o}{}^{2}}{V_{o}{}^{2}+I_{in}{}^{2}Z_{r}{}^{2}}\right)$$

**Figure 6:**
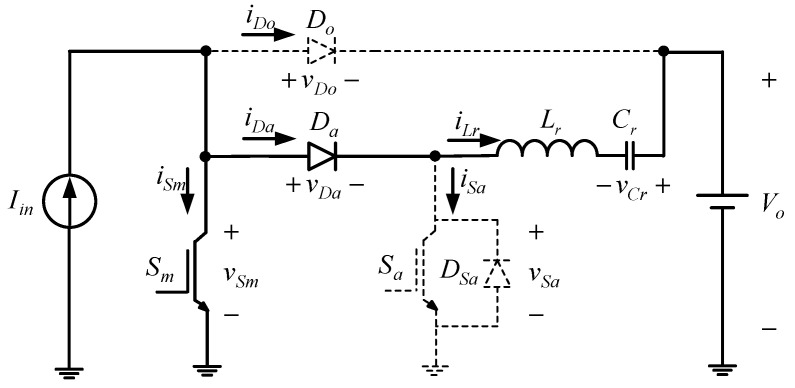
Current flow of state 4.

State 4 [$t_{3}\leq t\leq t_{4}$]: As shown in Figure 3 and Figure 6, when the time reaches *t*_3_, the auxiliary switch is cut off, and the resonant inductance *L_r_* and resonant capacitance *C_r_* continue to resonate, making the auxiliary diode conductive. At the same time, the resonant inductance current *i_Lr_* starts to rise. According to Kirchhoff’s current law, it can be known that ${Ι}_{in}=i_{Sm}+i_{Lr}$. As the resonant inductance current *i_Lr_* rises to the input current *I_in_*, the main switch current *i_Sm_* drops to zero. At this time, if the main switch is turned off, the ZCT of the main switch can be realized, and this state ends.

The initial conditions of this state are

(9) $$\left\{ \begin{array}{l} i_{Lr}(t_{3})=0\\ v_{Cr}(t_{3})=2V_{o}-v_{Cr}(t_{0}) \end{array}\right.$$

According to Figure 3, the following equations can be obtained as

(10) $$\left\{ \begin{array}{l} i_{Lr}(t)=\frac{V_{o}-v_{Cr}(t_{0})}{Z_{r}}\sin\omega_{r}(t-t_{3})\\ v_{Cr}(t)=V_{o}+\left[V_{o}-v_{Cr}(t_{0})\right]\cos\omega_{r}(t-t_{3}) \end{array}\right.$$

The time experienced in this state, called Δ*t*_4_, is

(11) $$\Delta t_{4}=t_{4}-t_{3}=\frac{1}{\omega_{r}}\sin^{-1}\left(\frac{Z_{r}I_{in}}{V_{o}-v_{Cr}(t_{0})}\right)$$

**Figure 7:**
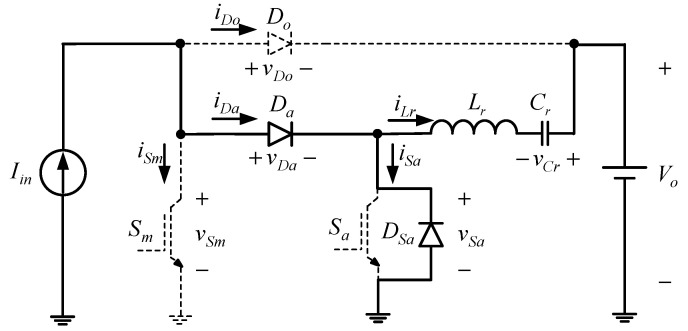
Current flow of state 5.

State 5 [$t_{4}\leq t\leq t_{5}$]: As shown in Figure 3 and Figure 7, when the time reaches *t*_4_, the main switch *S_m_* is cut off. At the same time, the resonant inductance *L_r_* and resonant capacitance *C_r_* continue to resonate. According to Kirchhoff’s current law, it can be known that ${Ι}_{in}=i_{Da}=i_{Sa}+i_{Lr}$. Since the resonant inductance current *i_Lr_* is greater than the input current *I_n_*, the internal diode of the auxiliary switch, called *D_Sa_*, is conductive. The moment the resonant inductance current *i_Lr_* drops to the input current *I_in_*, the operating state enters state 6.

The initial conditions of this state are

(12) $$\left\{ \begin{array}{l} i_{Lr}(t_{4})=I_{in}\\ v_{Cr}(t_{4})=V_{o}+V_{o}\cos(\omega_{r}\Delta t_{4}) \end{array}\right.$$

According to Figure 3, the following equations can be obtained as

(13) $$\left\{ \begin{array}{ll} i_{Lr}(t)= & \frac{V_{o}\cos(\omega_{r}\Delta t_{4})}{Z_{r}}\sin\omega_{r}(t-t_{4})\\ \; & +I_{in}\cos\omega_{r}(t-t_{4})\\ v_{Cr}(t)= & V_{o}+\left[V_{o}\cos(\omega_{r}\Delta t_{4})\right]\cos\omega_{r}(t-t_{4})\\ \; & -Z_{r}I_{in}\sin\omega_{r}(t-t_{4}) \end{array}\right.$$

The time experienced in this state, called Δ*t*_5_, is

(14) $$\Delta t_{5}=t_{5}-t_{4}=\frac{1}{\omega_{r}}\sin^{-1}\left(\frac{\left[V_{o}+V_{o}\cos(\omega_{r}\Delta t_{4})\right]I_{in}Z_{r}}{{\left[V_{o}\cos(\omega_{r}\Delta t_{4})\right]}^{2}+I_{in}{}^{2}Z_{r}{}^{2}}\right)$$

**Figure 8:**
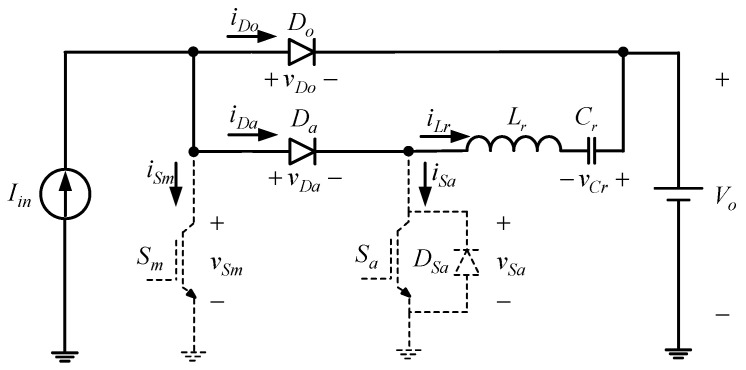
Current flow of state 6.

State 6 [$t_{5}\leq t\leq t_{6}$]: As shown in Figure 3 and Figure 8, when the time reaches *t*_5_, the resonant inductor *L_r_* and resonant capacitor *C_r_* resonate continuously, thus causing the resonant inductor current *i_Lr_* to drop from the input current *I_n_*. According to Kirchhoff’s current law, it can be known that $I_{in}=i_{Do}+i_{Lr}$. The resonant inductor current *i_Lr_* is smaller than the input current *I_n_*, thereby making the internal diode of the auxiliary switch, called *D_Sa_*, cut off and the output diode *D_o_* conductive. As soon as the resonant inductor current *i_Lr_* drops to zero, the operating state enters state 7.

The initial conditions of this state are

(15) $$\left\{ \begin{array}{l} i_{Lr}(t_{5})=I_{in}\\ v_{Cr}(t_{5})=0 \end{array}\right.$$

According to Figure 3, the following equations can be obtained as

(16) $$\left\{ \begin{array}{l} i_{Lr}(t)=I_{in}\cos\omega_{r}(t-t_{5})\\ v_{Cr}(t)=-Z_{r}I_{in}\sin\omega_{r}(t-t_{5}) \end{array}\right.$$

The time experienced in this state, called Δ*t*_6_, is

(17) $$\Delta t_{6}=t_{6}-t_{5}=\frac{1}{\omega_{r}}\sin^{-1}\left(\frac{-v_{Cr}(t_{0})}{Z_{r}I_{in}}\right)$$

**Figure 9:**
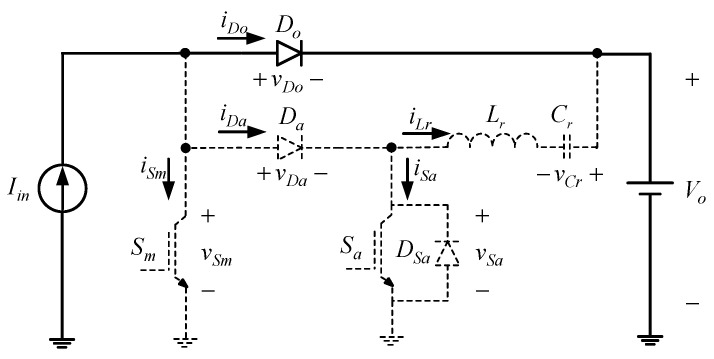
Current flow of state 7.

State 7 [$t_{6}\leq t\leq t_{7}$]: As shown in Figure 3 and Figure 9, when the time reaches *t*_6_, the resonance of resonant inductor *L_r_* and resonant capacitor *C_r_* is over; the resonant inductor current *i_Lr_* has dropped to zero, so the auxiliary diode *D_a_* is cut off, which is like the demagnetization state of the input inductor of the traditional boost converter.

According to Figure 3, the following equations can be obtained as

(18) $$\left\{ \begin{array}{l} i_{Lr}(t)=0\\ v_{Cr}(t)=v_{Cr}(t_{0}) \end{array}\right.$$

The time experienced in this state, called Δ*t*_6_, is

(19) $$\Delta t_{7}=t_{7}-t_{6}=(1-D)T_{s}-\Delta t_{5}-\Delta t_{6}$$

## 3. Proposed Current Sharing Strategy

**Figure 10:**
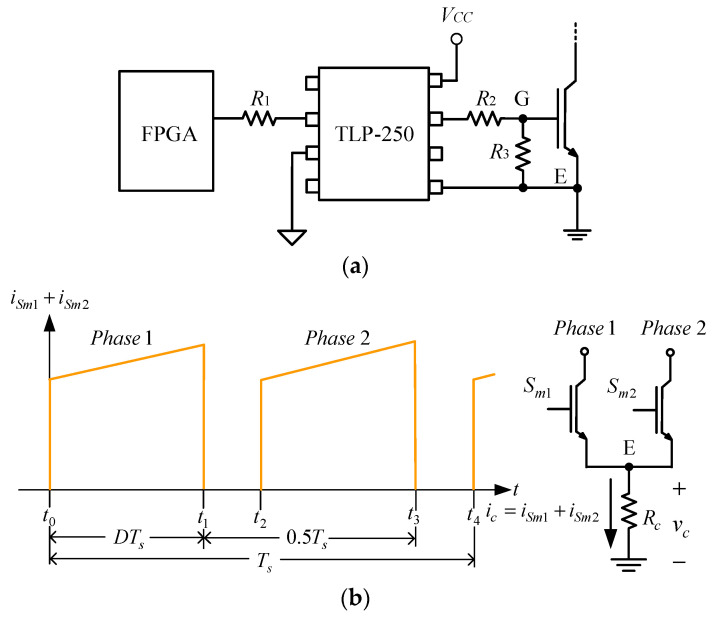
Proposed sampling method: (**a**) Relationship between TLP250 and IGBT; (**b**) Relationship between two TLP250s and *R_c_*.

Since the proposed system uses the interleaved PWM technique, the two phases have a 180° difference in their individual gate driving signals and do not overlap their individual duty cycles with each other. Accordingly, a new current sampling method is adopted herein. In this paper, we use a new current sampling method to obtain two-phase current signals with only one current sampling circuit, which reduces one current sampling device, one filter circuit, and one analog-to-digital converter (ADC). Figure 10 shows the proposed two-phase current sampling method, where the sampling device for the two-phase current, the main switch current of the first phase, and the main switch current of the second phase are shown. In this system, a current sampling resistor *R_c_* is connected in series with the emitters of the main switches of two phases. Based on the digital controller, the first-phase current is sampled from *t*_0_ to *t*_1_ time, and the second-phase current is sampled from *t*_2_ to *t*_3_ times so that the information of the two-phase current signals can be obtained.

### 3.1. Design of Current Sensing Resistor R_c_

Prior to this section, the system specifications will be given as shown in Table 1.

#### 3.1.1. Maximum Input Inductor Current

Since this converter operates in CCM from light load to rated load, the corresponding duty cycle *D* is shown as below:

(20) $$D=1-\frac{V_{i}}{V_{o}}=1-\frac{250}{400}\;=\;0.375$$

The input inductor current ripple Δ*i_Li_*_1_ can be obtained as follows:

(21) $$\Delta i_{Li1}=\frac{V_{i}DT_{s}}{L_{i1}}=\frac{250\times 0.375\times 20\mu }{1.66m}≅1.13A$$

The maximum input inductor current *I_L_*_1*,max*_ can be obtained as follows:

(22) $$I_{Li1,max}=I_{Li1}+\frac{1}{2}\Delta i_{Li1}=2+\frac{1}{2}\times 1.13≅2.57A$$

#### 3.1.2. Current Sensing Resistance

Figure 10a shows the isolated gate driver TLP-250 connected between gate G after resistor *R*_2_ and emitter E of the IGBT, whereas Figure 10b shows the current sensing resistor *R_c_* for the two phases. From these two figures, it is noted that since the low-level output voltage of the gate driver is connected to point E, the current in the gate driver does not flow through *R_c_*, which will guarantee that the IGBT works in the saturation region with *V_CE_*_(*sat*)_ smaller than 2.5 V, which is verified by experiment. The following will talk about how to obtain the value of *R_c_*.

Since the used current sensing resistor *R_c_* has the loss, this loss due to *R_c_* is set at 0.04% of the rated output power, that is, the power dissipated on *R_c_* is 400 mW. The current, due to the gate driver, does not flow through *R_c_* because the low-level output voltage of the gate driver is connected to point E.

According to Figure 10b, the rms value of the current flowing through *R_c_* is

(23) $$\begin{array}{ll} I_{c,rms}= & \sqrt{\frac{1}{0.5T_{s}}\int_{0}^{DT_{s}}i_{Sm1}{}^{2}dt}=\\ \; & \sqrt{2(I_{Li1,max}{}^{2}-2I_{Li1,max}\Delta i_{Li1}+\Delta i_{Li1}{}^{2})D+\frac{2}{3}\Delta i_{Li1}{}^{2}D}=3.935A \end{array}$$

Based on (23), two 0.1 Ω resistors connected in parallel are used as the current sensing resistor, where each resistor has a power dissipation of 200 mW, corresponding to the prescribed power dissipation. Since the maximum current flowing through *R_c_* is equal to *I_L_*_1*,max*_, the maximum voltage on *R_c_* is 0.1285 V. In addition, the maximum input voltage of the ADC is 5 V. Therefore, the voltage gain of the differential amplifier is set at 20, and hence, the ratio of voltage to current is 1 V/1 A and the maximum voltage on *R_c_* is 2.57 V, corresponding to the ADC requirement.

#### 3.1.3. Current Sharing Controller

**Figure 11:**
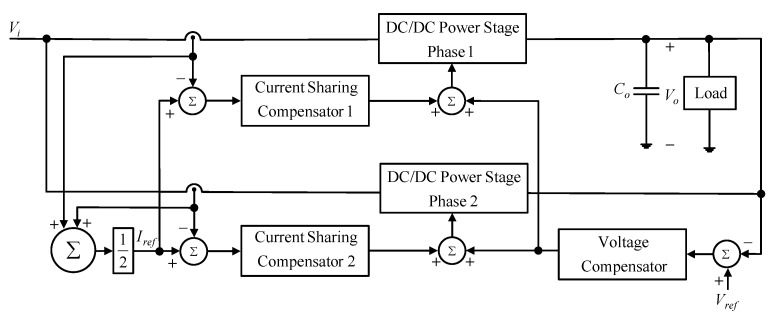
Block diagrams of the average current method.

In this paper, the average current method is used to make the input current evenly distributed between the two phases, as shown in Figure 11. For the average current method to be considered, the sampled current signals of two phases are averaged to generate the current sharing reference command *I_ref_*. Sequentially, each phase current is subtracted from this reference command to generate the current error signal and then feeds this error signal to the corresponding current sharing compensator to obtain the required control force. After this, this control force is added with the voltage control force created from the voltage compensator to control the corresponding switch of each phase, so that current sharing, as well as output voltage stabilization, can be achieved.

## 4. Design of Resonant Components

This section introduces the resonant component design according to the given system specifications, the resonant inductors and resonant capacitors of two phases are designed. The system specifications are shown in Table 1. First, the on-time of the auxiliary switch should be given, and after this, the values of the resonant components will be calculated under the rated condition as the worst condition. Accordingly, the ZCT operation under the light condition can be guaranteed, and this can be verified by measured waveforms, to be seen later.

**Figure 12:**
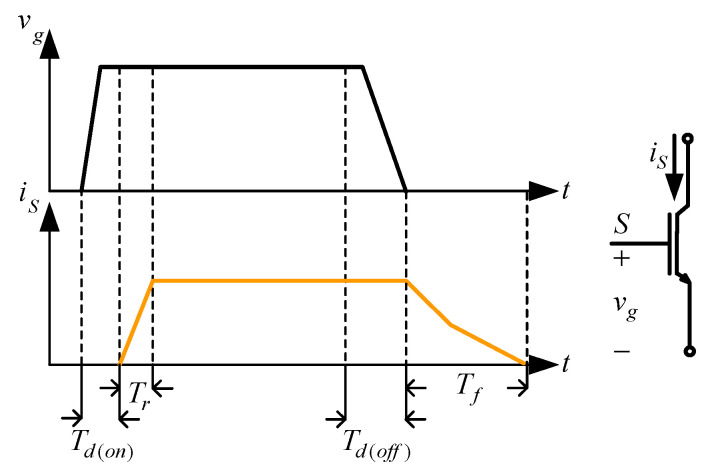
IGBT on delay time and off delay time.

From Sec. 2, the turn-on time of the auxiliary switch is used to determine the resonance time. If the auxiliary switch conduction time is too long, it is easy to cause an excessive increase in conduction loss, whereas if the turn-on time of the auxiliary switch is less than the sum of the cut-off delay time *T_d_*_(*off*)_ and falling time *T_f_* of the IGBT, the resonance inductor *L_r_* and the resonance capacitor *C_r_* will resonate several times and affect the operation of the auxiliary circuit. According to Figure 12, the on-time of the auxiliary switch must be greater than the sum of *T_d_*_(*off*)_ and *T_f_*. Therefore, to reduce the effect of the resonance frequency on PWM switching, the resonance period is set between 0.05 times the switching period and 0.1 times the switching period, that is, the resonance period locates between 1 μs and 2 μs, so the turn-on time of the auxiliary switch is taken as 1.5 μs.

According to Figure 3, since the resonant inductor and resonant capacitor resonate with two times the turn-on time of the auxiliary switch, the resonant angular frequency can be obtained to be

(24) $$\omega_{r}=2\pi \times f_{r}=2\pi \times \frac{1}{2\times 1.5\mu \;}=2.1M\mathrm{rad}/s$$

To ensure that the main switch can reach turn-off ZCT from light load to rated load, the peak current of the resonant inductor must be larger than the peak current of the input inductor, and when one phase is broken, the other phase must continue to operate. Therefore, the peak current of the resonant inductor is twice the peak current of the input inductor, and the resonant impedance can be obtained as

(25) $$2I_{Li1,max}=\frac{V_{o}-v_{Cr}(t_{0})}{Z_{r}}\Rightarrow Z_{r}=\frac{V_{o}-v_{Cr}(t_{0})}{2I_{Li1,max}}$$

As shown in Figure 3, the following equation can be approximately obtained to be

(26) $$\frac{v_{Cr}(t_{0})}{Z_{r}I_{Li1,max}}=\frac{Z_{r}I_{Li1,max}}{v_{Cr}(t_{0})-V_{o}}$$

Substituting (22) and (25) into (26) yields the initial resonant capacitance across the voltage as

(27) $$v_{Cr}(t_{0})=\frac{6V_{o}-\sqrt{36V_{o}{}^{2}+12V_{o}{}^{2}}}{6}=-62V$$

By substituting (25) into (26) and (27), the values of the resonant inductor and resonant capacitor can be obtained to be

(28) $$\left\{ \begin{array}{l} L_{r}=\frac{Z_{r}}{\omega_{r}}≅40\mu H\\ C_{r}=\frac{1}{\omega_{r}Z_{r}}≅5\mathrm{nF} \end{array}\right.$$

As shown in Figure 3, the maximum voltage across the resonant capacitor is 2*V_o_*-*v_Cr_*_(*t*_0_)_, i.e., 862 V. Therefore, the resonant capacitor takes a 1.5 kV Mylar capacitor, and the resonant inductor adopts a Sendust core manufactured by CHANGSUNG, with the model number CS270125, 18 turns, and the wire diameter No. 16 AWG.

## 5. System Configuration

**Figure 13:**
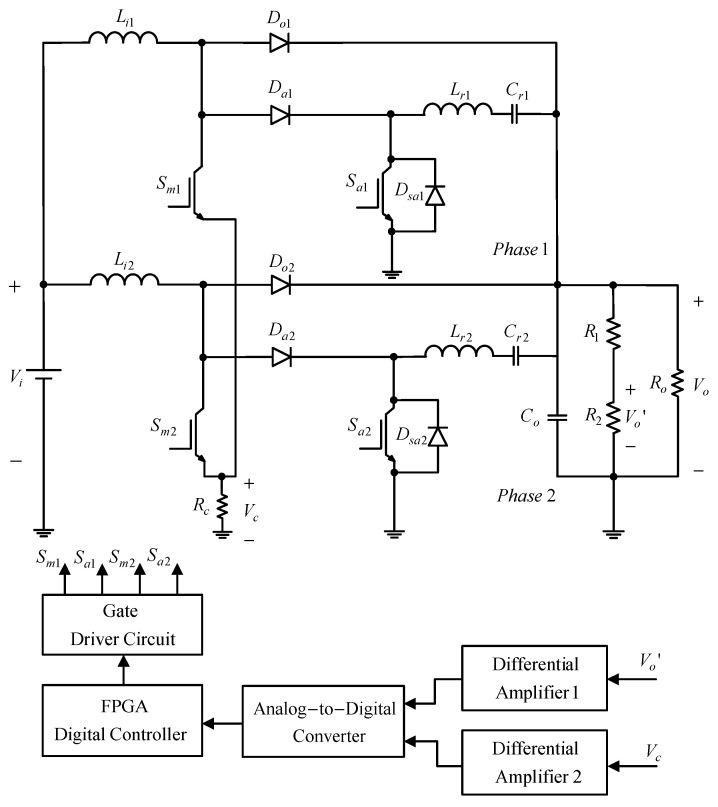
System circuit configuration.

In Figure 13, the main power stage is composed of a two-phase interleaved step-up converter with a ZCT auxiliary circuit, whereas the peripheral circuit includes a gate driver circuit, a voltage sampling circuit, a two-phase current sampling circuit and an analog-to-digital converter; in the controller, the FPGA is used as the control kernel to realize the fully digital interleaved PWM control. The Cyclone II series FPGA chip, manufactured by Altera Co., has a product number of EP2C20F484C8.

## 6. Simulated and Experimental Results and Discussions

### 6.1. Simulated and Measured Waveforms

**Figure 14:**
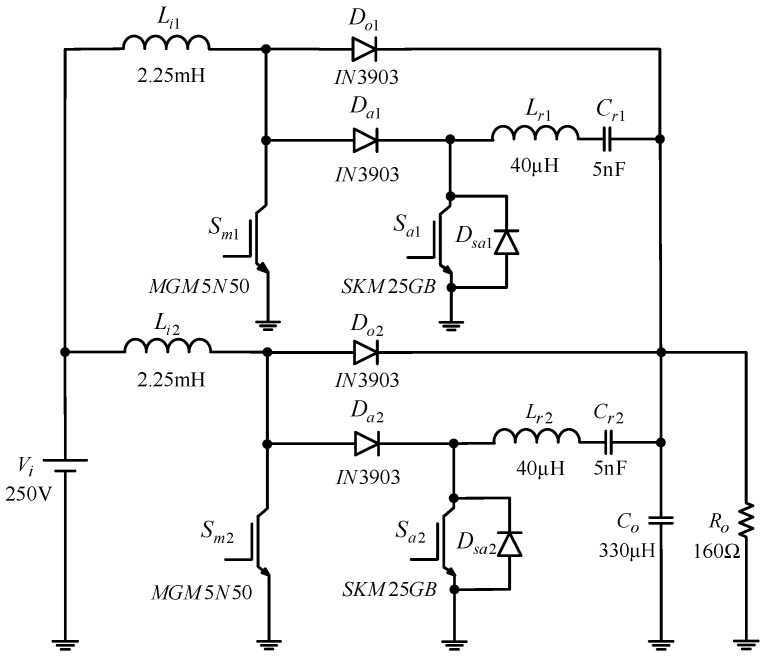
IsSpice simulation of the proposed circuit with selected component models and parameters.

The simulation shown in Figure 14 is based on the IsSpice software. In the following, the simulated and experimental results are obtained at rated load. Moreover, in Table 2, the part numbers used for the switches and diodes in the simulation and experiment can be configured according to the system specifications and the main operating waveforms.

**Figure 15:**
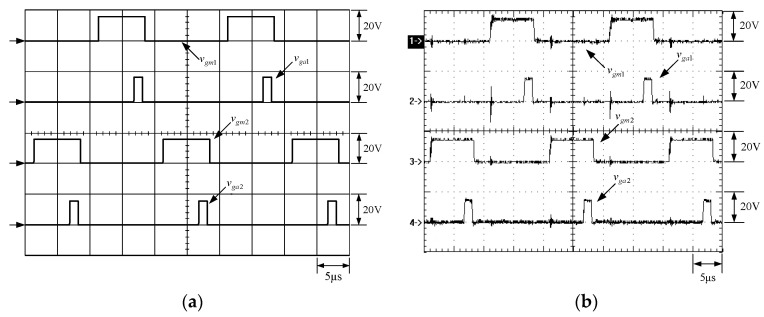
Waveforms relevant to gate driving signals: (1) *v_gm_*_1_; (2) *v_ga_*_1_; (3) *v_gm_*_2_; (4) *v_ga_*_2_ for (**a**) simulation and (**b**) experiment.

**Figure 16:**
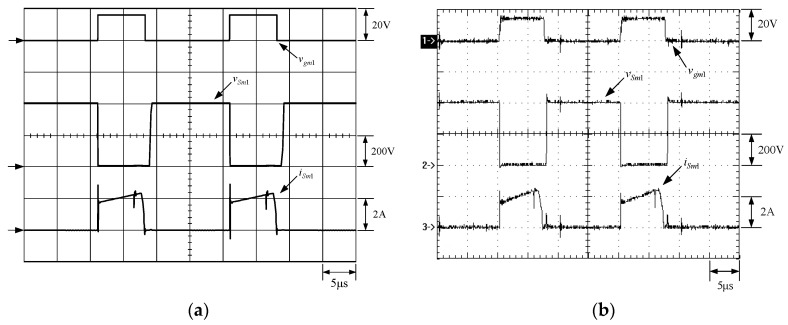
Waveforms relevant to *S_m_*_1_: (1) *v_gm_*_1_; (2) *v_Sm_*_1_; (3) *i_Sm_*_1_ for (**a**) simulation and (**b**) experiment.

**Figure 17:**
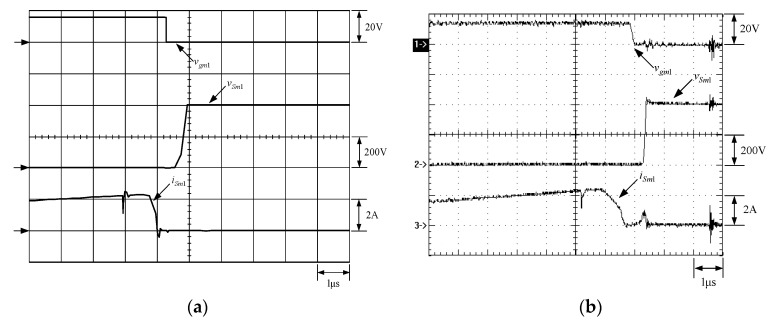
Zoom-in of Figure 17: (1) *v_gm_*_1_; (2) *v_Sm_*_1_; (3) *i_Sm_*_1_ for (**a**) simulation and (**b**) experiment.

**Figure 18:**
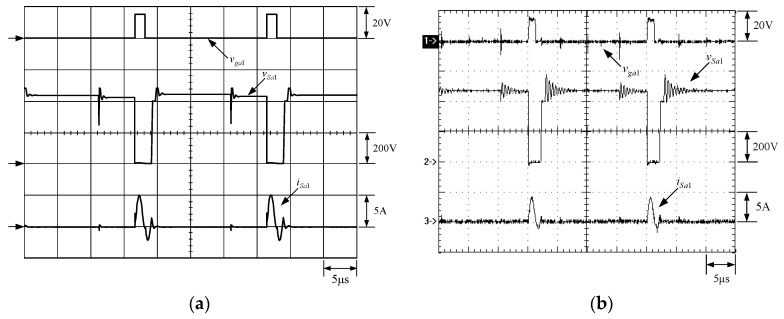
Waveforms relevant to *S_a_*_1_: (1) *v_ga_*_1_; (2) *v_Sa_*_1_; (3) *i_Sa_*_1_ for (**a**) simulation and (**b**) experiment.

**Figure 19:**
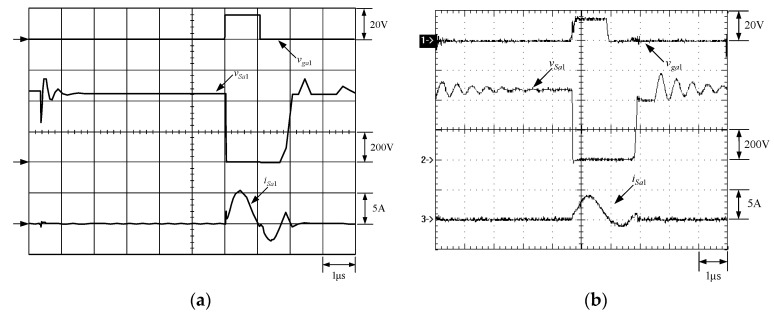
Zoom-in of Figure 19: (1) *v_ga_*_1_; (2) *v_Sa_*_1_; (3) *i_Sa_*_1_ for (**a**) simulation and (**b**) experiment.

**Figure 20:**
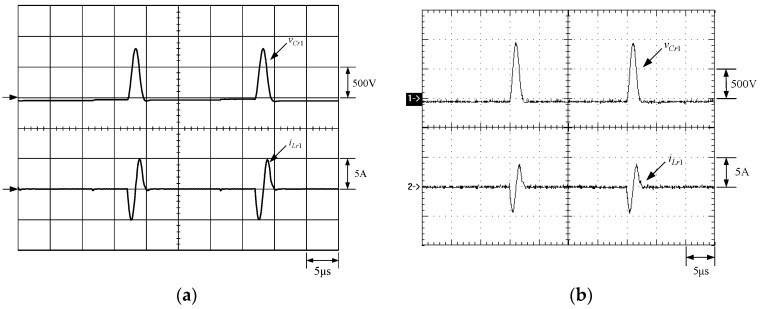
Waveforms relevant to *C_r_*_1_ and *L_r_*_1_: (1) *v_Cr_*_1_; (2) *i_Lr_*_1_ for (**a**) simulation and (**b**) experiment.

Figure 15 shows the gate driving signals for main switches *v_gm_*_1_ and *v_gm_*_2_, and auxiliary switches *v_ga_*_1_ and *v_ga_*_2_. Figure 16 shows the gate driving signal for the phase-1 main switch *S_m_*_1_, called *v_gm_*_1_, the voltage across *S_m_*_1_, called *v_Sm_*_1_, and the current flowing through *S_m_*_1_, called *i_Sm_*_1_. Figure 17 shows the zoom-in of Figure 16. Figure 18 shows the gate driving signal for the phase-1 auxiliary switch *S_a_*_1_, called *v_ga_*_1_, the voltage across *S_a_*_1_, called *v_Sa_*_1_, and the current flowing through *S_a_*_1_, called *i_Sa_*_1_. Figure 19 shows the zoom-in of Figure 18. Figure 20 shows the voltage on the resonant capacitor, called *v_Cr_*_1_, and the current flowing through the resonant inductor, called *i_Lr_*_1_.

**Figure 21:**
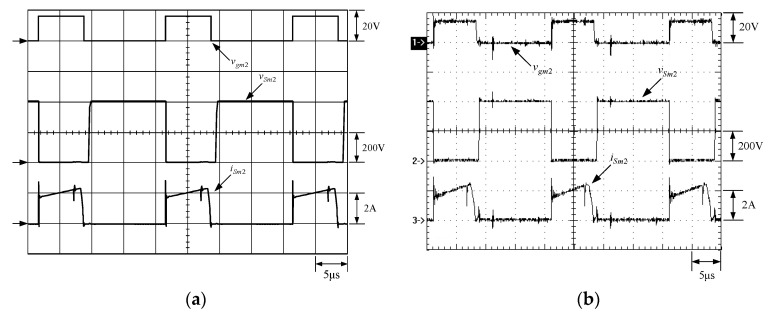
Waveforms relevant to *S_m_*_2_: (1) *v_gm_*_2_; (2) *v_Sm_*_2_; (3) *i_Sm_*_2_ for (**a**) simulation and (**b**) experiment.

**Figure 22:**
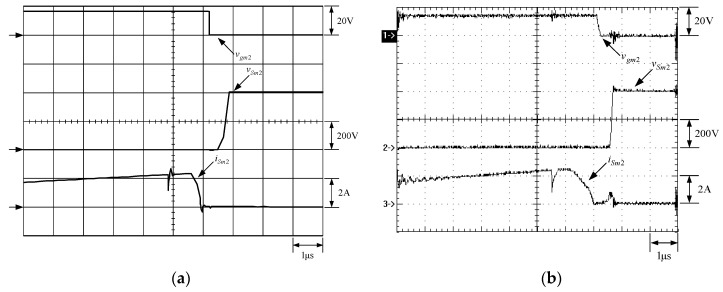
Zoom-in of Figure 23: (1) *v_gm_*_2_; (2) *v_Sm_*_2_; (3) *i_Sm_*_2_ for (**a**) simulation and (**b**) experiment.

**Figure 23:**
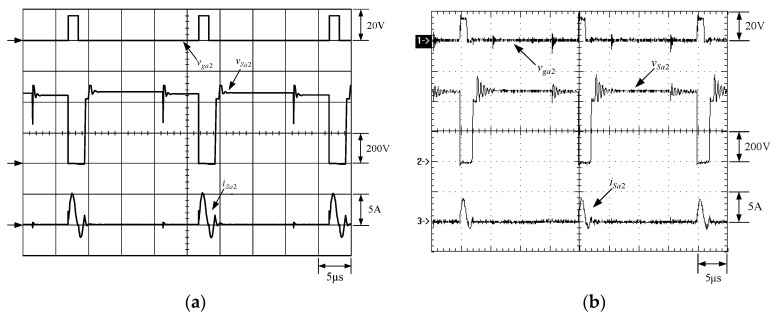
Waveforms relevant to *S_a_*_2_: (1) *v_ga_*_2_; (2) *v_Sa_*_2_; (3) *i_Sa_*_2_ for (**a**) simulation and (**b**) experiment.

**Figure 24:**
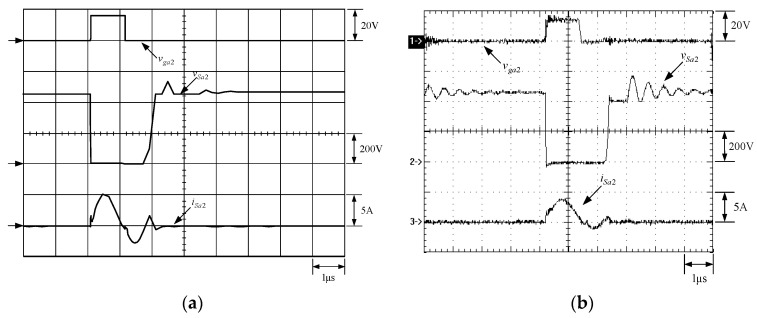
Zoom-in: (1) *v_ga_*_2_; (2) *v_Sa_*_2_; (3) *i_Sa_*_2_ for (**a**) simulation and (**b**) experiment.

**Figure 25:**
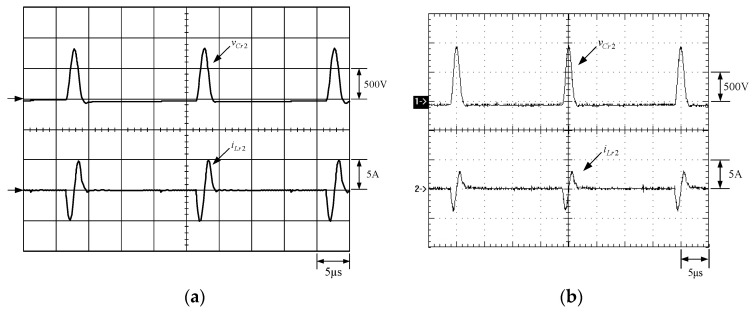
Waveforms relevant to *C_r_*_2_ and *L_r_*_2_: (1) *v_Cr_*_2_; (2) *i_Lr_*_2_ for (**a**) simulation and (**b**) experiment.

**Figure 26:**
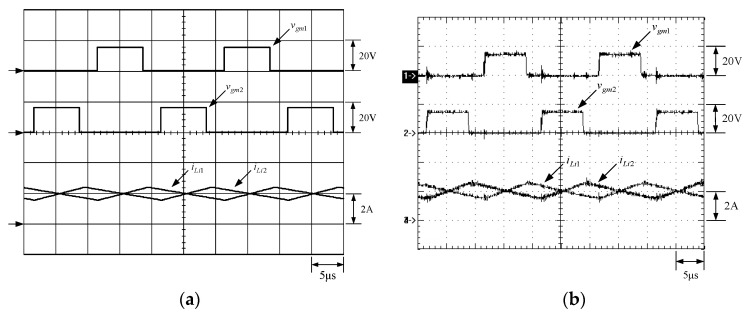
Waveforms relevant to *L_i_*_1_ and *L_i_*_2_: (1) *v_gm_*_1_; (2) *v_gm_*_2_; (3) *i_Li_*_2_; (4) *i_Li_*_2_ for (**a**) simulation and (**b**) experiment.

Figure 21 shows the gate driving signal for the phase-2 main switch *S_m_*_2_, called *v_gm_*_2_, the voltage across *S_m_*_2_, called *v_Sm_*_2_, and the current flowing through *S_m_*_2_, called *i_Sm_*_2_. Figure 22 shows the zoom-in of Figure 21. Figure 23 shows the gate driving signal for the phase-2 auxiliary switch *S_a_*_2_, called *v_Sa_*_2_, called *v_ga_*_2_, the voltage across *S_a_*_2_, called *v_Sa_*_2_, and the current flowing through *S_a_*_2_, called *i_Sa_*_2_. Figure 24 shows the zoom-in of Figure 23. Figure 25 shows the voltage on the resonant capacitor, called *v_Cr_*_2_, and the current flowing through the resonant inductor, called *i_Lr_*_2_. Figure 26 shows the gate driving signals *v_gm_*_1_ and *v_gm_*_2_, and the resonant inductor currents *i_Lr_*_1_ and *i_Lr_*_2_.

### 6.2. Waveform Comments

The simulated and experimental results are similar to some extent except for oscillation. From Figure 15, *v_gm_*_2_ is shifted by 180 degrees from *v_gm_*_1_ whereas *v_ga_*_2_ is shifted by 180 degrees from *v_ga_*_1_. Figure 17 shows *S_m_*_1_ has turn-off ZCT. Figure 19 shows *v_Sa_*_1_ and *i_Sa_*_1_, corresponding to near ZCS. Figure 20 shows *v_Cr_*_1_ and *i_Lr_*_1_, also corresponding to Figure 3. Figure 22 shows *S_m_*_2_ has turn-off ZCT. Figure 24 shows *v_Sa_*_2_ and *i_Sa_*_2_, corresponding to near ZCS. Figure 25 shows *v_Cr_*_2_ and *i_Lr_*_2_, also corresponding to Figure 3. Figure 26 shows *i_L_*_2_ is shifted by 180 degrees from *i_L_*_2_, the average values of the two currents are almost the same, meaning that the output current is almost distributed between the two phases.

In Figure 16 or Figure 21, there are four phenomena to be described. One is the current spike in *i_Sm_*_1_ or *i_Sm_*_2_ is due to the reverse recovery current of *D_o_*_1_ or *D_o_*_1_, and the voltage *v_Sm_*_1_ or *v_Sm_*_2_ during the turn-on period is smaller than the maximum on-voltage drop *V_CE_*_(*sat*)_ of 2.5 V based on the STGP10NC60H datasheet, thereby making sure that *S_m_*_1_ or *S_m_*_2_ are operated in the saturation region. Another is that *i_Sm_*_1_ or *i_Sm_*_2_ will have a small increase when the voltage across *S_m_*_1_ or *S_m_*_2_ rises since the IGBT needs time to withdraw injected carriers at the emitter of the IGBT during the turn-off period. The other is that as *S_a_*_1_ or *S_a_*_2_ is on instantaneously, the corresponding *D_a_*_1_ or *D_a_*_2_ is on, so some of the currents in *S_m_*_1_ or *S_m_*_2_ will be shifted to *S_a_*_1_ or *S_a_*_2_, resulting in a current dip in current *i_Sm_*_1_ or *i_Sm_*_2_.

In Figure 18 or Figure 23, there are two ringing voltages on *v_Sa_*_1_ or *v_Sa_*_2_ to be described. One is that since the reverse recovery current of *D_Sa_*_1_ or *D_Sa_*_2_ will flow to the resonant path, the first ringing voltage on *v_Sa_*_1_ or *v_Sa_*_2_ will occur. The other is that when *S_m_*_1_ or *S_m_*_2_ turn on instantaneously, the reverse recovery current of *D_o_*_1_ or *D_o_*_2_ will flow to the resonant path through *D_a_*_1_ or *D_a_*_2_, so the second ringing voltage on *v_Sa_*_1_ or *v_Sa_*_2_ will occur.

**Figure 27:**
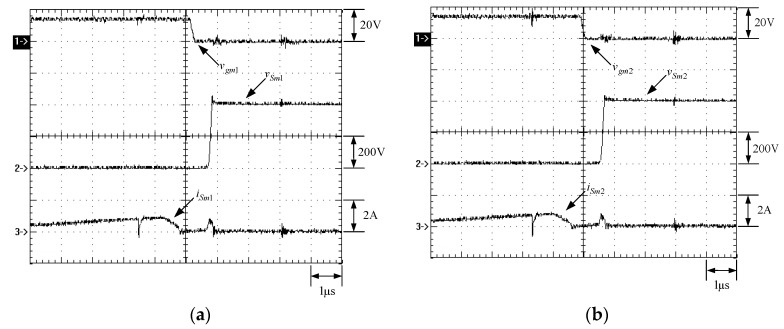
Waveforms under 25% load: (1) *v_gm_*_1_; (2) *v_Sm_*_1_; (3) *i_Sm_*_1_ for (**a**) *S_m_*_1_; (1) *v_gm_*_2_; (2) *v_Sm_*_2_; (3) *i_Sm_*_2_ for (**b**) *S_m_*_2_.

In Figure 20 or Figure 25, the peak value of the resonant inductor current is smaller than the designed value due to the presence of parasitic resistance in the actual circuit, and the peak value of the resonant capacitor voltage is larger than the designed value due to the tolerance of the resonant capacitance. From Figure 27, it is noted that at light load, the main switches *S_m_*_1_ and *S_m_*_2_ still have ZCT turn-off, corresponding to the requirements.

### 6.3. Light-Load Efficiency Improvement

**Figure 28:**
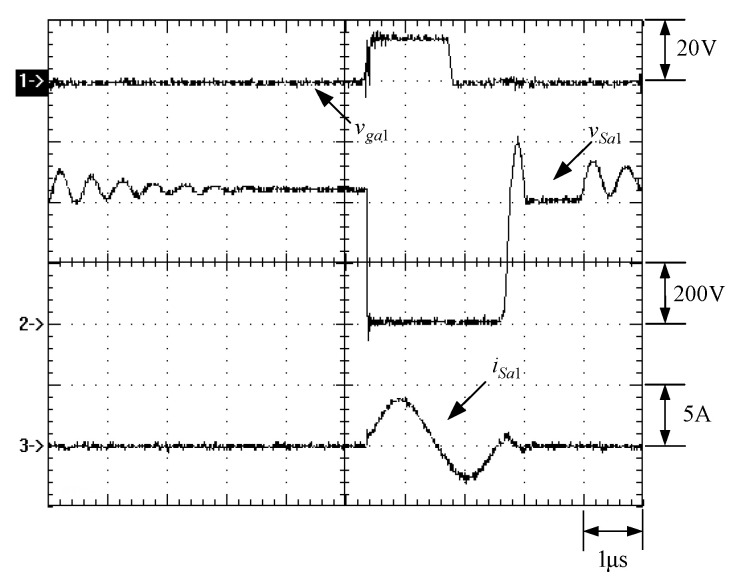
Waveforms related to phase-1 *S_a_*_1_ under 25% load without the on-time and off-triggering moment of *S_a_*_1_ adjusted: (1) *v_ga_*_1_; (2) *v_Sa_*_1_; (3) *i_Sa_*_1_.

**Figure 29:**
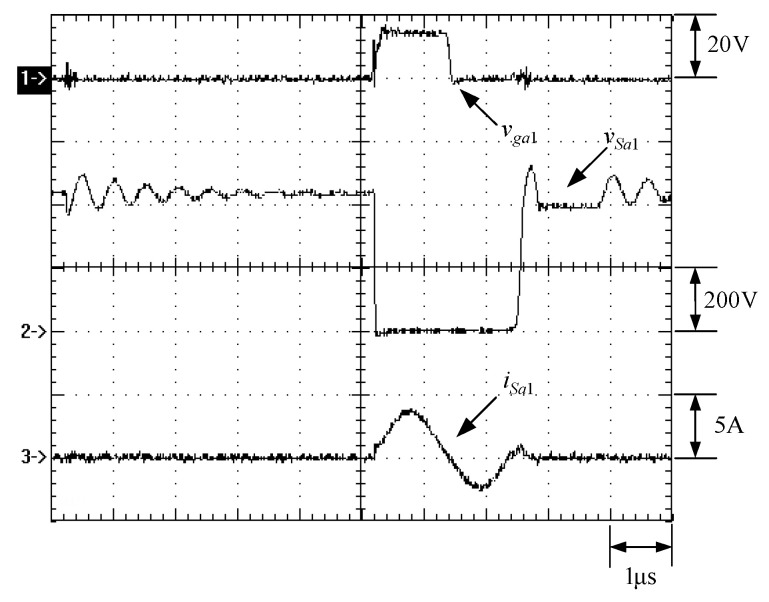
Waveforms related to phase-1 *S_a_*_1_ under 25% load with the on-time and off-triggering moment of *S_a_*_1_ adjusted: (1) *v_ga_*_1_; (2) *v_Sa_*_1_; (3) *i_Sa_*_1_.

Figure 28 shows the waveforms of *S_a_*_1_ under 25% load by not adjusting the on-time and off-triggering moment of *S_a_*_1_. As can be seen from this figure, when the proposed converter is operated under a light load, the current flowing through *D_sa_*_1_ is larger than that under a rated load. Therefore, the reverse recovery current of this diode is larger than that under a rated load, so a voltage spike on *S_a_*_1_ is likely to be caused. Figure 29 shows the waveforms of *S_a_*_1_ under 25% load by adjusting the on-time and off-triggering moment of *S_a_*_1_. The peak value of the voltage spike will be reduced from 620 V to 460 V, thereby making the light-load efficiency improved. This description can be applied to phase-2 *S_a_*_2_.

### 6.4. Efficiency Measurement

**Figure 30:**
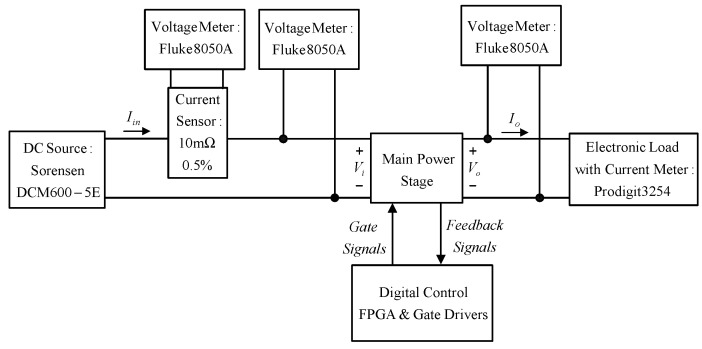
Efficiency measurement block diagram.

**Figure 31:**
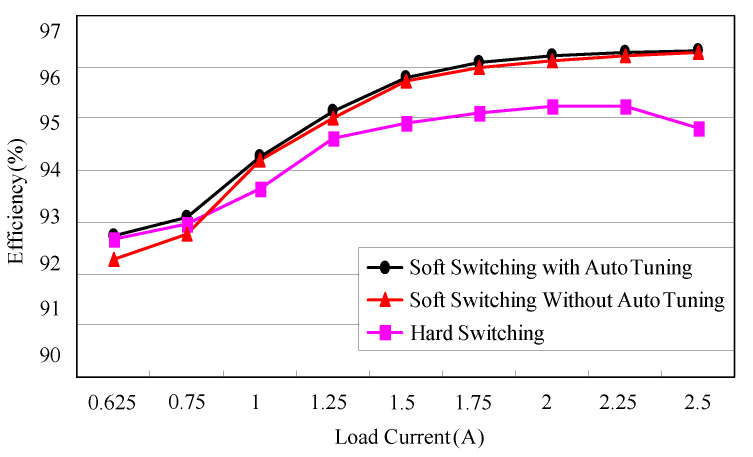
Curves of efficiency versus load current.

Figure 30 shows the efficiency measurement block diagram. From this figure, a current sampling resistor is connected in series with the input current path. The input current and input voltage are multiplied to obtain the input power. To measure the output power, an electronic load, named Prodigit 3254, is used to provide the required load current, and a digital voltmeter, named Fluke 8050 A, is used to measure the output voltage, which is multiplied by the load current to obtain the output power. Eventually, the output power at each load is divided by the corresponding input power to obtain a curve of efficiency versus load current shown in Figure 31. In Figure 31, there are three cases to be compared. Case 1 has the ZVT turn-on without the on-time and the off-triggering instant of *S_a_*_1_ and *S_a_*_2_ adjusted. Case 2 has the ZVT turn-on with the on-time and the off-triggering instant of *S_a_*_1_ and *S_a_*_2_ adjusted. Case 3 has no ZVT. From this figure, Case 2 has better light-load efficiency than the other two. The rated load efficiencies for Case 1, Case 2 and Case 3 are 96.3%, 96.4% and 94.8%, respectively. Since the power loss relevant to the tail current is quite difficult to estimate [24], we use the rated-load efficiencies for Case 3 and Case 1 to figure out the individual power losses of 54.8 W and 38.4 W, respectively, and then the power loss associated to the falling current can be approximately estimated out by the difference in power loss between these two cases, equal to 16.4 W.

### 6.5. Comparison between the Existing and the Proposed

In Table 3, the literature [14] has the least number of main switches, and the main switches have a hard turn-on and ZCS turn-off; however, the main switches will have large current stresses when they are turned off. In [9], the main switches have a large voltage spike when they are turned off. Additionally, although the auxiliary switches have turn-off ZVS and ZCS, they are turned off with higher voltage surges so that the components with higher voltage withstand are needed. The output diode, although it has a turn-on near ZCS and turn-off ZVZCS in [9], there are high resonance voltages and resonance currents, resulting in the need for a higher voltage and current-withstanding components for the output diode. The auxiliary diode has a soft switching function in every literature except for [14]. The maximum efficiency is the highest in [13]; however, the average efficiency of [13] is not the highest. Although the maximum efficiency of the proposed circuit is not the highest, its average efficiency has been improved by using an auto-tuning technique, and hence the practicality of this circuit has been greatly increased.

## 7. Conclusions

This paper proposes a fully digital multiphase interleaved boost converter with a fixed-frequency ZCT. The proposed auxiliary circuits used to realize the ZCT with the common-grounded auxiliary switches are added to the traditional boost converter to reduce the switching loss caused by the hard switching of IGBTs. In addition, the overall efficiency of the converter can be improved by adjusting the on-time and off-trigger times of the auxiliary switch. Furthermore, a current-sharing control along with a two-phase interleaved control is used to distribute the input current evenly among the phases. In addition, a new current sampling method is used, which requires only one sampling circuit to simultaneously return the current signals of the two-phase main switches. Above all, the number of phases can be extended, still possessing easy control of the ZCT and current sharing.

## Figures and Tables

**Table 1:** System specifications.

Specification	Value or Condition
Input Voltage (*V_i_*)	250 V
Output Voltage (*V_o_*)	400 V
Rated Output Current (*I_o,rated_*)/Rated Output Power (*P_o,rated_*)	2.5 A/1 kW
Min. Output Current (*I_o,min_*)/Min. Output Power (*P_o,min_*)	0.625 A/0.25 kW
Switching Frequency (*f_s_*)/Switching Period (*T_s_*)	50 kHz/20 μs
Operating Mode	Continuous Current Mode (CCM)
Input Inductance	1.66 mH
Output Voltage Ripple	Smaller than 0.15% of *V_o_*

**Table 2:** Part numbers used for the switches and diodes on simulation and experiment.

	Switch and Diode	Part Number
Simulation	Main Switches *S_m_*_1_ and *S_m_*_2_	STGP10NC60H
	Aux. Switches *S_a_*_1_ and *S_a_*_2_	STGP10NC60HD
	Output Diodes *D_o_*_1_ and *D_o_*_2_	IN3903
	Aux. Diodes *D_a_*_1_ and *D_a_*_2_	IN3903
Experiment	Main Switches *S_m_*_1_ and *S_m_*_2_	MGM5N50
	Aux. Switches *S_a_*_1_ and *S_a_*_2_	SKM25GB
	Output Diodes *D_o_*_1_ and *D_o_*_2_	BYV29
	Aux. Diodes *D_a_*_1_ and *D_a_*_2_	BYV29

**Table 3:** Comparison of the existing and the proposed.

Ref.	Switch	Main Switch		Aux. Switch		Output Diode		Aux. Diode		Max. Eff.
		On	Off	On	Off	On	Off	On	Off	
[9]	4	Hard	ZCS	Near ZCS	ZVS ZCS	ZVS	ZCS ZVS	ZVS ZCS	Near ZCS	96.7%
[13]	4	Hard	ZCS	Near ZCS	ZCS	ZCS	Hard	Hard	Near ZCS	98.5%
[14]	3	Hard	ZCS	Near ZCS	ZCS	ZVS	ZCS	-	-	92.2%
[15]	4	Hard	ZCS	Near ZCS	ZCS	Near ZCS	Near ZCS	ZCS	ZVS	95.5%
[16]	6	Hard	ZCS	Near ZCS	ZCS	Near ZCS	Near ZCS	Near ZVSNear ZCS	Hard	97.6%
[17]	4	Hard	ZCS	Near ZCS	ZCS	Near ZCS	Near ZCS	ZVS ZCS	ZCS ZVS	98%
Proposed	4	Hard	ZCS	Near ZCS	ZCS	Near ZCS	Hard	ZVS	Near ZCS	96.4%

## Data Availability

No new data were created or analyzed in this study. Data sharing is not applicable to this article.
